# Frontiers in diagnostic and therapeutic approaches in diabetic sensorimotor neuropathy (DSPN)

**DOI:** 10.3389/fendo.2023.1165505

**Published:** 2023-05-18

**Authors:** Sanjeev Sharma, Gerry Rayman

**Affiliations:** Department of Diabetes and Endocrinology, Ipswich Hospital, East Suffolk and North East Essex NHS Foundation Trust (ESNEFT), Ipswich, United Kingdom

**Keywords:** diabetes, polyneuropathy, small fibre neuropathy, large fibre neuropathy, early diagnosis diabetes neuropathy

## Abstract

Diabetes sensory polyneuropathy (DSPN) is a significant complication of diabetes affecting up to 50% of patients in their lifetime and approximately 20% of patients suffer from painful diabetes neuropathic pain. DSPN – both painless and painful - leads to considerable morbidity including reduction of quality of life, increased lower limb amputations and is associated with worsening mortality. Significant progress has been made in the understanding of pathogenesis of DSPN and the last decade has seen newer techniques aimed at its earlier diagnosis. The management of painful DSPN remains a challenge despite advances made in the unravelling the pathogenesis of pain and its transmission. This article discusses the heterogenous clinical presentation of DSPN and the need to exclude key differential diagnoses. Furthermore, it reviews in detail the current diagnostic techniques involving both large and small neural fibres, their limitations and advantages and current place in the diagnosis of DSPN. Finally, the management of DSPN including newer pharmacotherapies are also discussed.

## 1 Introduction

The term diabetes sensorimotor polyneuropathy (DSPN) refers to a heterogeneous group of neurological disorders – either clinically evident or subclinical – that occur in the setting of diabetes mellitus and cannot be attributed to other aetiologies of peripheral neuropathy (1). It is by far the he commonest form of diabetes polyneuropathy (DPN) (**Table 1**) affecting up to 50% of people with diabetes, while its yearly incidence amounts to approximately 2% (3, 4). DSPN is defined as a symmetrical, length-dependent polyneuropathy attributable to not only chronic hyperglycaemia-mediated microvascular alterations but also contributed by other cardiovascular factors including dyslipidaemia, hypertension and smoking (1).

**Table 1:** Classification of diabetes neuropathy [adapted from Pop-Busui et al. (2)].

I. DIFFUSE NEUROPATHY		
**A. Distal symmetrical polyneuropathy (DSPN)**	
	i) Primary large fibre involvement
	ii) Primarily small fibre involvement
	iii) Mixed large and small fibre involvement (commonest)
**B. Autonomic neuropathy**	
	**i) Cardiovascular** *Resting tachycardia* *Orthostatic hypotension* *Reduced heart-rate variability* *Malignant arrhythmias*
	**ii) Gastrointestinal** *Diabetes gastropathy* *Diabetes enteropathy* *Colonic dysmotility*
	**iii) Urogenital** *Erectile dysfunction* *Neurogenic bladder* *Female sexual dysfunction*
	**iv) Sudomotor dysfunction** *Gustatory sweating* *Distal hypohydrosis*
	**v) Hypoglycaemic unawareness**
	**vi) Pupillary abnormalities**
II. Mononeuropathy (mononeuritis multiplex)		
**A. Isolated cranial nerve (III/VI) palsy**	
**B. Peripheral nerve palsies (ulnar, median, femoral, peroneal)**	
**C. Mononeuritis multiplex**	
III. Radiculopathy or polyradiculopathy		
**A. Radiculoplexus neuropathy (lumbosacral radiculopathy, diabetes amyotrophy)**	
**B. Thoracic radiculopathy**	

The classification of DSPN varies and is based on a number of factors including presence or absence of painful symptoms, pattern of neural involvement and the setting of its evaluation – clinical or research. Chronic painful DSPN is found in up to 25% of subjects with diabetes and is defined as persistent or recurrent pain lasting 3 months and caused by a lesion or disease of the somatosensory system due to diabetes and after exclusion of other causes (5, 6). (**Table 2**) The position statement by American Diabetes association (ADA) proposed a clinical classification of DSPN into either primarily large and small fibre or mixed based on the type of neural fibres involved (2). This differentiation will be more elaborately discussed later.

**Table 2:** Common non-diabetes neuropathies that need to be excluded whilst diagnosing DSPN.

**I. **Chronic inflammatory demyelinating polyneuropathy
**II. **Pressure palsies
**III. **Neuropathies caused by alcohol abuse, uraemia, vitamin B12 deficiency, paraproteinaemias, hypothyroidism, cancer and infectious diseases and neurotoxic drugs
**IV. **Neurotoxic drugs (platinum analogues, taxanes, vinca alkaloids etc.)
**V. **Radiculoplexus neuropathy
**VI. **Acute painful small fibre neuropathy (e.g. acute insulin neuritis syndrome)

**Figure 1:**
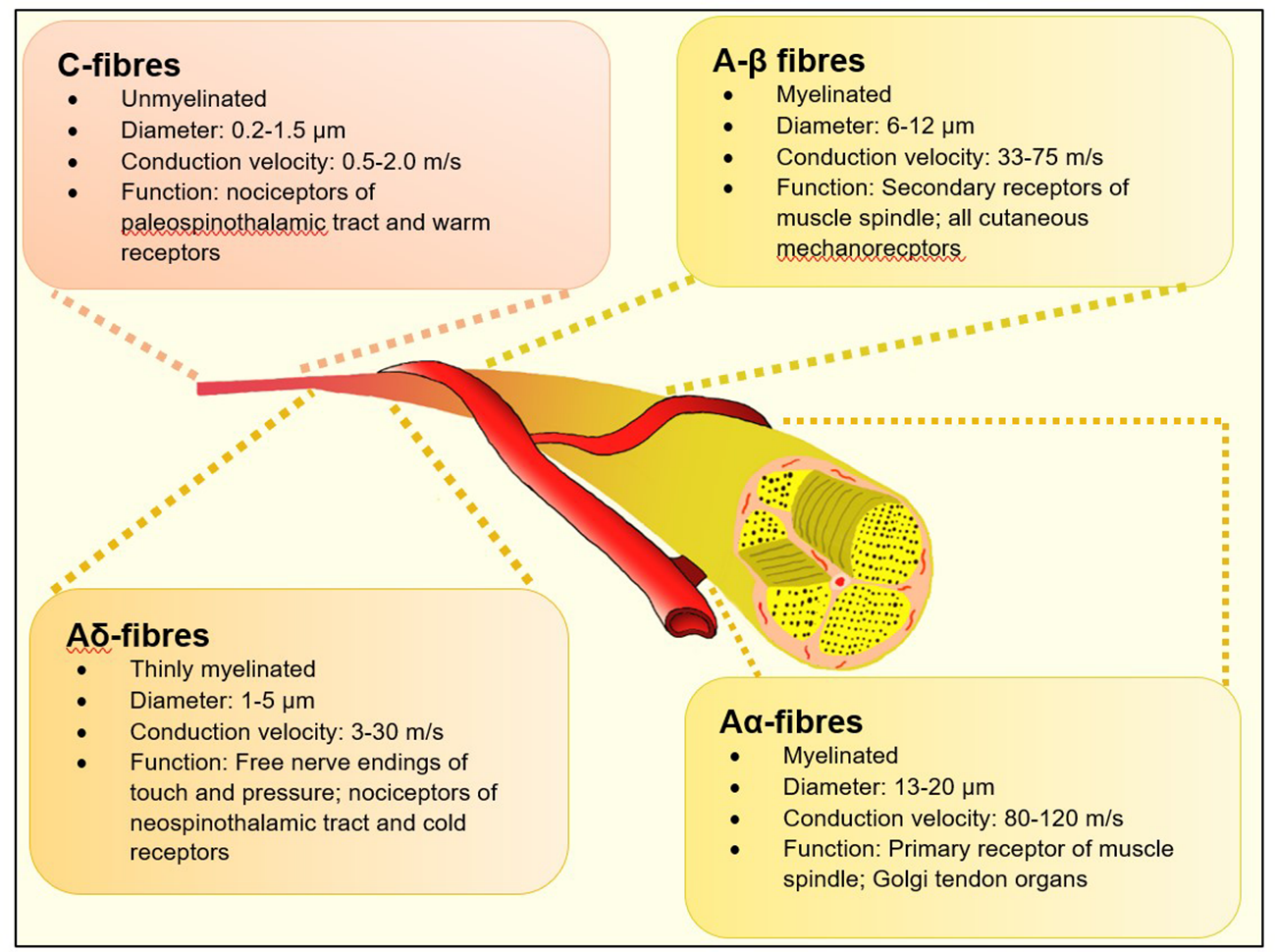
Types of neural fibres [adapted from Sharma S et al. (9)].

A practical definition of DSPN in the clinical setting is the presence of neuropathic symptoms as reported by the patient and/or signs of peripheral nerve dysfunction in diabetes subjects after exclusion of other causes. Historically – in busy diabetes clinics – the presence of abnormal ankle reflex, abnormal 128 Hz tuning fork sensation or insensitivity to 10-gm Semmes-Weinstein monofilament (SWMF) was considered to be valid evidences to diagnose DPN. More accurate assessments like nerve conduction studies were time consuming and resource-specific and hence reserved for atypical presentation including truncal radiculopathy, entrapment neuropathy and amyotrophy (7, 8). Whist these methods were undoubtedly useful in assessing risk of patients of foot ulceration, they are limited in their capabilities to assess mostly Aα and Aβ nerve myelinated fibres (**Figure 1**) which serve touch, proprioception, position sense, vibration and muscle control. Consequently, they are affected later in the natural history of diabetes (10). There is now a large body of evolving evidence that small neural fibres (thinly myelinated Aδ and unmyelinated C fibres) are affected early in the natural history of DSPN and precedes large fibre involvement (11). Hence the specific assessment of this group offers hope for earlier diagnosis of DSPN which can lead to quicker adoption of disease modifying strategies such as life style modification, glycaemic, blood pressure and lipid control or novel therapeutics to prevent onset and progression of worsening neuropathy (9).

## 2 Clinical presentation of DSPN

The commonest clinical presentation of DSPN is a distal symmetrical type of progressive sensorimotor polyneuropathy which is length dependent i.e., the lower limbs are affected earlier than upper extremities and with progression of the condition, there is distal to proximal axonal degeneration; hence many patients present with the typical “stocking and glove” distribution of symptoms (2). Up to 50% of subjects remain asymptomatic and hence the need for at least annual review of their feet sensations to prevent ulceration. Symptoms at presentation can be either “negative” (loss of sensations including touch, vibration, position or deficits including ataxia) or “positive” (pain, paraesthesia, allodynia). Not uncommonly patients report the subjective symptoms of “numbness” which could indicate either of the above group of symptoms and it is up to the clinicians to make this differentiation to enable treatment (12). It is important to note that autonomic dysfunction is common in patients with DSPN but is largely undetected unless heralded by symptoms as mentioned in **Table 1** (2).

Common determinants for progression of DSPN include patient characteristics (advancing age, height and obesity), clinical parameters (presence of hypertension and uncontrolled dyslipidaemia) and adverse glycaemic matrices (poor glycaemic control, longer duration of diabetes, beta cell insufficiency) (1, 3, 13). Whilst it is beyond the scope of this article to examine each of these determinants individually, research into early diabetes neuropathy using methods of small fibre function have shown that the impact of glycaemic control in the progression of microangiopathy is much more important in type 1 diabetes whilst in type 2 diabetes, the impact is more multifactorial (14, 15).

## 3 Assessment of DSPN

### 3.1 Bedside foot screening

The traditional and still mostly prevalent screening tool assessment of diabetes feet in busy clinical environments remains the use of a 10gm-SWMF or 128-Hz tuning form (detects large fibres) and pin-prick sensation (detects small fibres) (2). It must be highlighted that these bedside techniques are useful for assessing diabetes feet at risk of future ulceration and are not sensitive for early detection of DSPN (16). More recently, the Ipswich Touch test has been shown to be an equally sensitive method for evaluating at-risk feet with a strong concordance with the 10-gm monofilament (17, 18).

The diagnosis of painful DSPN is based on presence of painful neuropathic symptoms and a clinical diagnosis of DSPN after exclusion of other neuropathies as shown in **Table 2**.

### 3.2 Use of Scored clinical assessments

Over the years, many scored clinical assessments have been proposed as standardized objective and quantitative measures for screening and grading of severity of DSPN. Their utility is particularly useful for epidemiological studies looking at prevalence of DSPN in larger populations. Of these, the Michigan Neuropathy Screening Instrument (MNSI) (2, 19), Toronto Clinical Neuropathy Score (20), modified Toronto Neuropathy Score (21), Neuropathy Disability Score (16), Neurological Disability Score (22), Neuropathy Symptom Score (22) and Utah Early Neuropathy (23) are commonly used. It is beyond the scope of this article to examine each score individually but their usage is limited by the time required in busy clinical settings. Recently, machine-learning severity prediction tools based on the MNSI have been proposed to save time (24).

For painful DSPN, useful scoring tools include the Neuropathy Total Symptom Score-6 (25), DN4 (26), PainDETECT (27) and Neuropathic Pain Symptom Inventory (28).

### 3.3 Rapid point-of care devices

In order to overcome the shortcomings of screening tools and to reduce the time taken by scoring systems in clinical practice, a number of point-of-care devices have evolved in the past few years which have the ability to reduce bias and provide more accurate results. The Vibratip™ for objective assessment of vibration sense is one such device (29). Another device is DPNCheck™ which a hand-held device providing sural nerve conduction velocity and amplitude in under five minutes correlates well with Neuropathy Disability score (30, 31). Similarly the SUDOSCAN™ is another well-established device which by assessing electrochemical skin conductance as a measure sudomotor function has been shown to show early small fibre changes (autonomic system) in patients at risk of DSPN (32, 33).

### 3.4 Large fibre methodologies

i) The neurothesiometer is a battery-operated device that provides mechanical vibration with a fixed frequency of approximately 100 Hz while the vibration amplitude is controlled manually using a rotatory control knob. The knob is used to adjust the voltage applied and ranges from 0 to 50 V (0-250 µm in amplitude). The operator applies the handheld probe to the pulp of the great toe and the vibration stimulus gradually increased, until the subject feels the vibration sensation. The voltage displayed on the neurothesiometer is the measured vibration perception threshold (VPT). It has been shown to more consistent than vibration sense and hence reflective of peripheral nerve function; hence it is commonly used in clinical research trials (34). The major drawback of such a device is its manual observer-dependent operability and its limited vibration intensity (35, 36).
ii) Nerve conduction studies (NCS) measure properties of transmission of electrical current along nerve and muscle fibre membranes. The nerves tested in diabetes for DSPN include both lower and upper limb peripheral nerves which include motor and sensory nerve. The first NCS abnormality observed in DSPN is a reduction of sural nerve amplitude and as the condition progresses, the distal sensory latency increases and finally there is reduction of conduction velocity (37).
One of the major criticisms of NCS in DSPN is that only large fibre activity is measured which is known to be affected in later stages (9). Furthermore, the normative ranges for NCS parameters overlap with abnormal values in DSPN limiting the sensitivity and accuracy of testing. Also, NCS must be corrected for anthropomorphic factors such as gender and body mass index to improve their accuracy (38). Despite these concerns, NCS remains an important objective measure of neuropathy and is still used to differentiate DSPN from other atypical aetiologies.
iii) Electromyography in DSPN is supplementary and exploratory to NCS above. It is of value when there are atypical presentations like radiculopathy, pure motor myopathy or inflammatory myopathy. Percutaneous needle insertion into muscles in required; a procedure that can be uncomfortable and hence not well suited for serial studies (37).

### 3.5 Small fibre methodologies

Small fibre neuropathy (SFN) is a sub-type of peripheral neuropathy affecting the thinly myelinated Aδ or the non-myelinated C fibres (**Figure 1**). These fibres constitute 79.6% to 91.4% (39, 40), of peripheral nerve fibres and mediate several key functions including temperature and pain perception, sweating, and tissue blood flow - all of which, when impaired are pathophysiologically related to adverse outcomes associated with foot ulcerations in people with diabetes

Small fibre methods can be broadly classified into methods for assessing small fibre function and small fibre structure. **Table 3** provides a summary of methods currently available.

**Table 3:** Overview of the current methodologies for testing small fibres in DSPN [adapted from Sharma S et al. (9)].

Functional tests for SFN									
Test	Type	Technique			Equipment Needed	Time to acquire results	Normative Data	Operating Characteristics for DSPN	Limitations
**Quantitative sensory testing (QST)** (41)	Non-invasive, quantitative	Computerised measurement of thermal thresholds and heat pain thresholds			Computerised assessment device, temperature controlled laboratory and a trained technician	Takes about half an hour but could take longer, depending on subject concentration	Commercial normative data present from the bigger manufacturers.	None available	Psychophysical test- results are dependent on subject compliance and attention. Complex testing protocols present. Varying reproducibility depending on experience of the unit undertaking testing.
**Laser Doppler imager Flare (LDIflare)** (42)	Non-invasive, quantitative (**Figure 2**)	Measurement of the axon-reflex mediated flare response as a marker of small fibre function			Laser Doppler imager, temperature controlled room, operator with experience	Image acquisition took ~1 hour with the older method. Newer method takes approximately 25 minutes. Results available immediately	One site normative values determined at a single centre. Larger data set of normative valves desired (43)	For the newer technique: Sensitivity of 70-75%, specificity of 66-85%, positive predictive value of 74%, and negative predictive value of 86%	Dependent on the microcirculation. Patients need to have no significant macrovascular distal circulatory impairment. Has correlation with Confocal microscopy (44).
**Current perception threshold (CPT)** (45)	Non-invasive, quantitative	Low current intensity stimulation of the small nerve fibres at frequency of 250 Hz for A-delta fibres and the 5 Hz for C-fibres.			Neurometer device temperature controlled room and a trained technician	Takes about half an hour but could take longer, depending on subject concentration	None available. Most studies have included age matched controls for comparison.	None available	Requires active patient co-operation. Like QST, therefore reproducibility has been a challenge and other methodological challenges persist (such as what frequency to use. Not widely available.
**Contact Heat Evoked Potentials (CHEPs)** (46)	Non-invasive, quantitative	Measure cerebral responses to thermal stimuli mediated by A-delta fibres			Needs thermal threshold testing first. Then small discs are placed on the head to record signals received to the brain from application of 10 to 20 short (a fraction of a second) heat or cold stimuli at a particular point of interest (face, arm or leg)	Takes about half an hour but could take longer, depending on subject concentration	Multicentre normative data on 226 adult subjects are available	The AUC for DSPN detection in a small sample has been estimated at 0.778.	Requires active patient co-operation. Like QST and CPT, therefore reproducibility has been a challenge. Not widely available. Also unclear if both A-delta and C-fibres are assessed.
**Microneuro-graphy** (47)	Minimally invasive, semi quantitative	Measurement of Single fibre recordings from peripheral axons			Skilled operator and extensive equipment list. Preserve of a large neurophysiology lab rather than clinic based procedure.	May take up to 3 hours to get a satisfactory recording.	None available	None available. Considered by EFNS to possess grade A evidence for assessing function of the A-delta fibre pathways in patients with neuropathic pain	Still primarily a research tool. May have a role in assessment of neuropathic pain rather than early neuropathy. Expensive and needs skill to elicit responses. Patient cooperation is also extensively required.
**Laser Evoked Potentials (LEPs)** (48)	Non-invasive, quantitative	Radiant heat generate by laser selectively excites free nerve endings in the superficial skin layers activating nearby A-delta and C -fibre nociceptors			CO2-laser stimulator, technician with experience and a temperature controlled room ideally.	May take up to 1 hour to complete the procedure and ensure no artefacts presents in readings gained	Single centre normative values available on 100 subjects. No decade specific data reported.	None available. Studies have used age matched control data.	Limited availability. May be useful in demonstrating reduced function but unable to detect enhanced transmission as found in hyperalgesia. Small changes in pain sensitivity are not easily detectable with LEP
**Quantitative sudomotor axon reflex test (QSART)** (49)	Sudomotor Non-invasive, quantitative	Information on skin autonomic function and evaluation of postganglionic sudomotor function using acetylcholine iontophoresis		Purpose built lab, iontophoresis and sudomotor quantification equipment.		45-60 minutes to complete.	Normative data available from specific centres for QSART. A commercially available device QSWEAT is also available	No specific data available for DSPN but has been widely used, especially in the Rochester Diabetic Neuropathy study	Requires precautions for electrical safety and small risk of minor local injury to the skin
**Thermo-regulatory sweat test (TST)** (50)	Sudomotor Non-invasive, semi-quantitative	When core temperature rises beyond a hypothalamic thermoregulatory set point (>38°C), sweating occurs		Needs a laboratory and a digital camera		90-120 minutes to perform correctly. Maximal sweating is achieved within 30–65 minutes.	Unclear	Helpful data on the TST available in DSPN mainly from the autonomic lab at the Mayo Clinic, Rochester USA.	Patients may not be able to tolerate 60 minutes of warming up
**Sympathetic Skin Response** (51)	Sudomotor Non-invasive, quantitative	Information on skin autonomic function and evaluation of postganglionic sudomotor function using electrodermal activity		Purpose built lab, SSR equipment includes electrodes.		45-60 minutes to complete.	Normative data available from specific centres but usually has been derived from a small normative group	Minimal data only available in DSPN. Some helpful data in diabetic autonomic neuropathy and bladder dysfunction.	Limited availability, needs expertise and experience to test correctly. Popular in Japan.
**Sudoscan^®^** (32)	Sudomotor Non-invasive, quantitative	Testing is based on stimulation of sweat glands by a low-voltage current (<4volts) representing a electrochemical reaction between electrodes and chloride ions,		Just the Sudoscan^®^ device		Takes less than 5 minutes	Comes with inbuilt normative data. Limited experience at the moment	Increasing literature now available of its use in DSPN. Similar AUC as IENFD (0.761) in one study. For Cardiac autonomic neuropathy sensitivity was 65%, specificity 80%.	Still limited availability. Needs more detailed validation work for different ethnicities.
**Neuropad^®^** (52)	Simple qualitative indicator of sudomotor dysfunction	Simple sticker which changes colour in the presence of sweating.		Cheap and easy to avail.		Takes less than 10 minutes	Qualitative, does not need normative data.	Lots of available literature and has been validated against IENFD. In one study, Neuropad had a sensitivity 85% and specificity of 45% for detection of clinical DSPN.	Difficult to interpret when there is partial change in colour though. One centre has published data on semi-quantification using digital imaging of the Neuropad^®^.
Structural tests for SFN									
**Skin Biopsy** (53)	Invasive (minimally), quantitative	Measurement of intra-epidermal nerve fibre density	Sterile equipment for biopsy, access to trained personnel and laboratory			Procedure takes 5-10 minutes but takes a few days to get the results back.	Worldwide normative Data present	Published sensitivity doe DSPN is between 60% and 95% and specificity between 90% and 95%	Challenging to use in prospective studies of very large cohorts, infection risk at site of biopsy
**Sural nerve biopsy** (54)	Invasive, quantitative	Ultrastructure and morphometric analysis of sural nerve biopsy specimens	Experienced operator who can perform biopsy and access to pathologist and at times, electron or confocal microscope			Procedure may take up to 45 minutes. Results usually take a few days.	None available	None available	Infection, pain and hypoesthesia at biopsy site
**Corneal Confocal Microscopy** (55, 56)	Non-invasive, quantitative	Measurement of nerve parameters of the corneal sub-basal layer	Corneal scanning confocal microscope, trained technician			Image acquisition takes 5-10 minutes. Results available immediately if automated counting used	Worldwide normative Data present	Reported sensitivity of 85% and specificity of 84%	Surrogate marker of DSPN rather than a direct indicator. Previously reliant on manual counting but newer automated methods emerging. Unclear which of the three- CNFB, CNFL or CBNFD best representative/predictive of DSPN

**Figure 2:**
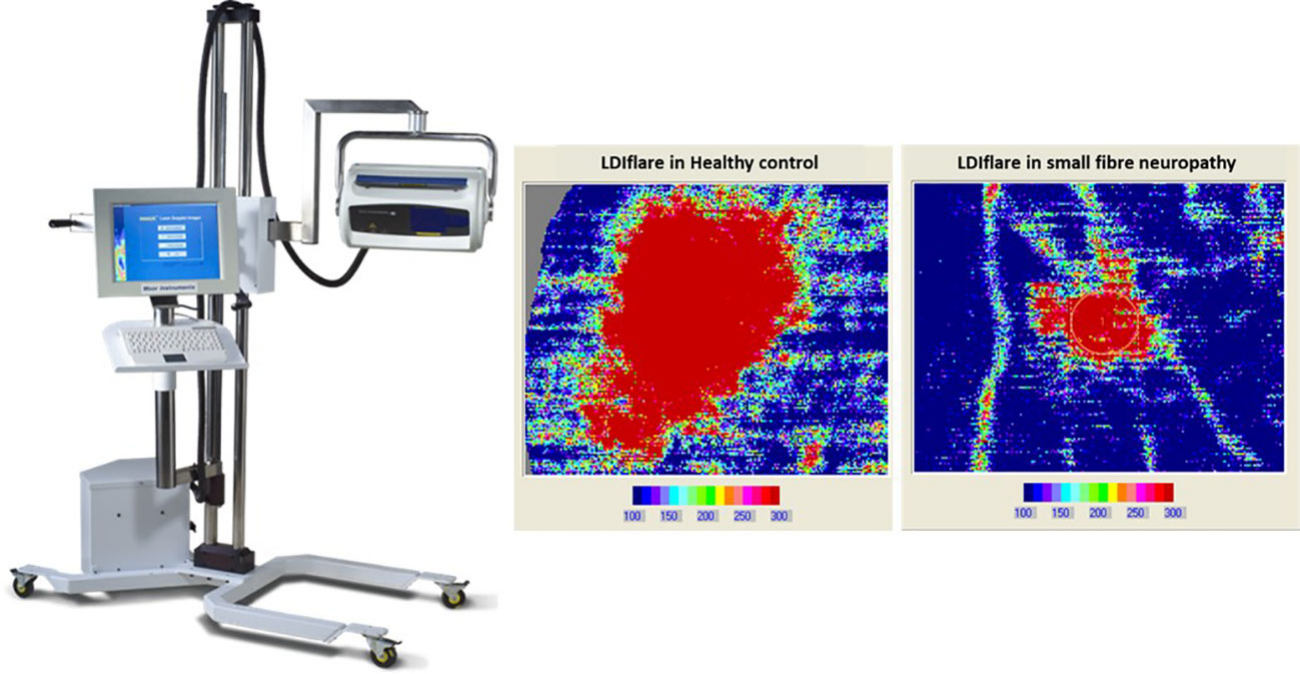
The laser doppler imager (LDI_flare_) is a non-invasive technique of measuring C fibre-mediated cutaneous vasodilatation in the foot skin in response to thermal heating. The heating is done by a 1cm2 probe and the area of induced hyperaemia measured by a 610nm laser probe. The scan image on left is a 11cm^2^ flare area in a healthy control while on the right is 1.1cm^2^ flare in a subject with small fibre neuropathy.

### 3.6 Tests for autonomic neuropathy

Autonomic dysfunction is common in up to 50% of subjects with DSPN but remains either asymptomatic or undiagnosed. The presenting features include impairment of cardiovascular, gastrointestinal, urogenital, thermoregulatory, sudomotor, and pupillomotor function. The accurate diagnosis of the autonomic neuropathies has been enhanced by the availability of physiological tests that measure autonomic function, and more recently, structural studies of the autonomic cutaneous innervation (57). Presence of cardiovascular autonomic neuropathy (CAN) in the presence of DSPN is an independent risk factor increased mortality and morbidity (58). The Composite Autonomic Symptom Score (COMPASS) 31 score is a validated, easy-to-use, quantitative assessment tool for autonomic symptoms in diabetic neuropathy, with a fair diagnostic accuracy for both cardiovascular autonomic neuropathy and diabetic polyneuropathy (59). A detailed description for autonomic neuropathy tests is beyond the scope of this article where the focus in mainly on DSPN.

### 3.7 Newer research methodologies

Both the brain and the spinal cord have been implicated in the genesis of painful DSPN and broadly involves a shift towards excitation and reduced inhibition of transmission of pain signals (60). The brain network involved in such chronic pain states is partially distinct from those involved in acute pain. Magnetic resonance spectroscopy has been used to determine the neuronal function in the thalamus and primary somatosensory cortex in patients with and without painful DSPN. Early results have shown thalamic neuronal dysfunction was found in only advanced painless DSPN but preserved in both subclinical and painful DSPN. These methods show promise in improving our understanding of painful DSPN and might have a role in future targeted pharmacotherapy (61).

## 4 The conflicting evidences supporting early use of small fibre methodologies for diagnosis of DSPN

The pillars of diabetes management include early diagnosis of complications and steps to prevent their worsening. For other microangiopathic complications, advances have been made for their early detection e.g., retinal screening for diabetes retinopathy and urinary microalbuminuria for diabetes nephropathy (62). Yet in contrast, The current ADA position statement on DPN endorses the following: “*Assessment for distal symmetric polyneuropathy should include a careful history and assessment of either temperature or pinprick sensation (small-fibre function) and vibration sensation using a 128-Hz tuning fork (for large-fibre function). All patients should have annual 10-g monofilament testing to identify feet at risk for ulceration and amputation”* (2, 4). It should be emphasized that the ADA’s position is to “*identify feet at risk for ulceration and amputation”* rather than early diagnosis of neuropathy; in essence the major focus of this guidance is the prevention of disabling diabetes lower limb complications.

A comprehensive review of various methodologies for the assessment of DSPN has been done but it is to be noted that none of them, except the screening bedside tools have found their way to any established guidance for detection of DPN. There are many reasons for this: firstly, the evidences available are mostly cross-sectional and only 6 studies have been performed longitudinally – the longest being at 5 years (63). Secondly, the lack of globally agreed-upon normative data for these tests make them less practical for routine use. So far, only confocal microscopy (CCM) and intra-epidermal nerve fibre density (IEFND) have acceptable normative values (64, 65). Thirdly, age-related decline in neural function and structure is well recognised. Hence normative data should also be age-linked and only the LDI_FLARE_ technique has shown such age-linked reduction of small fibre function (43).

Furthermore, it well known that with duration of diabetes, there is progression of other microangiopathic complications including diabetes kidney disease and it is well known that progressive uraemia affects neural health (66). Hence normative values should also be linked to renal health i.e. linked to measures of creatinine and eGFR. Finally, the methodologies of various techniques are still not uniform with various centers using their own adaptions to improve performance. Hence in view of considerable heterogeneity involved with various techniques, it is unlikely that they will appear in guidelines soon unless a much-needed consensus is agreed upon by investigators and equally importantly accepted by legislators.

## 5 Management of DSPN

The pillars of management of DSPN can be categorized as follows;

### 5.1 Reduction of risk factors contributing to progression of DSPN

Adoption of healthy lifestyle measures and weight loss remain the cornerstone of non-pharmacological management of DSPN. Whilst there are epidemiological studies which have shown the prevalence of less neuropathic symptoms in type 2 diabetes subjects with lower body weight (67). Interestingly, in a recent prospective cohort study of 131 obese participants attending a medical weight-management program, improvements were observed on the MNSI questionnaire, two Quality of Life in Neurological Disorders subdomains, and quantitative sensory testing cold thresholds whilst IENFD remained stable (68).

The impact of intensive glycaemic control in retarding the progression of DSPN has been well demonstrated in type 1 diabetes in the DCCT/EPIC study (69) but specific evidence to suggest the same is lacking in type 2 diabetes (70, 71). The reason for the latter could be due to the difference of pathogenesis of neuropathy in both types of diabetes and the effect of metabolic syndrome in type 2 diabetes. Nevertheless, it is well accepted that improved glycaemic control reduces the onset and progression of DSPN.

The EURODIAB IDDM study (13) of 3250 type 1 diabetes subjects showed significant correlations between the presence of diabetic peripheral neuropathy with age, duration of diabetes, quality of metabolic control, height, the presence of background or proliferative diabetic retinopathy, smoking, dyslipidaemia and the presence of cardiovascular disease. However prospective studies are lacking to indicate that control of any of the above factors are helpful in retarding the progression of DSPN although it is widely believed that hypertension and dyslipidaemia need to be aggressively managed to limit microangiopathic complications (72, 73).

### 5.2 Specific pharmacotherapy

Several compounds are available which target major pathways implicated in the pathogenesis of DSPN including polyol pathway, hexosamine pathway, protein kinase C (PKC) activity, and advanced glycation end products (AGEs) pathway (29). The AGE inhibitor Benfotiamine and antioxidant A-lipoic acid are licensed as drugs for treatment of DSPN in many countries (74, 75). In the NATHAN 1 trial, neuropathic deficits improved after 4 years in patients with mild to moderate largely asymptomatic DSPN (76). Actovegin, a poly(ADP-ribose) polymerase (PARP) inhibitor, is authorized in mainly eastern European countries, while the aldose reductase inhibitor Epalrestat is being used in India and Japan for DPN (77, 78).

Similarly for painful DSPN, several pharmacotherapies have been shown to reduce painful symptoms by their mechanisms of action on the pathways mentioned above. Treatment with A-lipoic acid 600mg twice daily for 6 months has been shown to reduce symptoms of pain, paresthesia and numbness in symptomatic DSPN (79). A-lipoic acid infusions over 3 weeks have also been shown to significantly reduce neuropathic symptoms (75). Larger randomized controlled trials are needed to study their efficacy in multicentered population subsets to strengthen the rationale of use in DSPN.

### 5.3 Symptomatic pharmacological treatment of painful DSPN

Painful DSPN can often be a crippling symptom leading to significant reduction of quality of life for patients, many of whom also have neuropathic deficits leading to complications. The management of painful DSPN is often challenging due to difference in efficacy between individual agents, balance between side-effects and working patterns and drug interactions. Monotherapy is usually not tolerated in maximal dosages and recent evidences from the OPTION-DM study showed that combination treatment was well tolerated and led to improved pain relief in patients with suboptimal pain control with a monotherapy (80, 81). The broad group of mediations in this class are shown in **Table 4**.

**Table 4:** Dosages and adverse events and pharmacotherapies used in the management of DSPN in clinical practice (82–86).

Drug	Class of drug	Daily dosage range	Common side effects
**Duloxetine**	serotonin-norepinephrine reuptake inhibitors	30 – 120 mg	somnolence, headache, nausea, dry mouth
**Amitriptyline**	tricyclic antidepressants	10 – 100 mg	somnolence, dizziness, headache, dysarthria, aggression, dry mouth, nausea, constipation, weight gain, hyperhidrosis
**Pregabalin**	a2δ calcium channel ligand	75 – 600 mg	somnolence, dizziness, headache
**Gabapentin**	a2δ calcium channel ligand	300 – 3600 mg	somnolence, dizziness, ataxia,
**Venlafaxine sustained release**	serotonin-norepinephrine reuptake inhibitors	75 – 225 mg	insomnia, dizziness, sedation, headache, nausea, dry mouth, constipation, hyperhidrosis
**Tapentadol extended release**	µ-opioid	50 – 200 mg	somnolence, vertigo, headache, nausea, emesis
**Oxycodone extended release**	µ-opioid	10 – 50 mg	somnolence, vertigo, headache, nausea, constipation
**Tramadol extended release**	µ-opioid	50 – 200 mg	vertigo, nausea
**Topical 8% Capsaicin patch**	transient receptor potential vanilloid agonist	Plaster applied for 30 min every 60–90 days	pain and erythema in site of application

### 5.4 Non-pharmacological treatment of painful DSPN

In diabetes subjects with refractory pain, spinal cord stimulation has been shown to significant pain relief and improved quality of life. It is an expensive invasive procedure needing insertion of percutaneous leads placed epidurally and connected to an implantable pulse generator typically placed in the low back. Whilst the mechanism of action is yet to fully understand, early results are promising but long-term results are awaited (87, 88). Other methods such as cognitive behavioral therapy, transcutaneous electrical nerve stimulation and acupuncture have a low level of evidence but can be successful in individual subjects (89).

## 6 Conclusion

DSPN is a major complication of diabetes. By the time a diabetes subject develops foot ulceration, the cardiovascular risk for death is increased by 50% and their 5-year mortality is worse than most cancers. With the worldwide prevalence of diabetes increasing to pandemic standards, it is anticipated that the consequences of DSPN in diabetes will continue to be on rise leading to serious challenges in both personal wellbeing and global health expenditure. Whilst on one hand, considerable advances in the understanding of the mechanisms of DSPN have been made, as discussed in this article, its early diagnosis and effective management remains uncertain and lacks uniformity. Whilst medications like the glucagon-like-peptide 1 receptor agonists and sodium-glucose co-transporters 2 have shown significant benefit in reducing cardiovascular mortality, heart failure and chronic kidney disease associated with diabetes, there is still a substantial unmet need in the therapeutics governing DSPN and other aspects of diabetes polyneuropathy. It is our combined responsibility to pool our resources, invest in uniform management algorithms and convince legislators to embrace newer diagnostic techniques so that burden and sequalae of DSPN can be lessened.

## Author contributions

SS and GR researched, wrote and reviewed all parts of this manuscript.

## References

[1] Tesfaye S Boulton AJM Dyck PJ Freeman R Horowitz M Kempler P. Diabetic neuropathies: update on definitions, diagnostic criteria, estimation of severity, and treatments. Diabetes Care (2010) 33:2285–93. doi: 10.2337/dc10-1303 PMC2945176 20876709

[2] Pop-Busui R Boulton AJ Feldman EL Bril V Freeman R Malik RA. Diabetic neuropathy: a position statement by the American diabetes association. Diabetes Care (2017) 40:136–54. doi: 10.2337/dc16-2042 PMC6977405 27999003

[3] Ziegler D Papanas N Vinik AI Shaw JE. Epidemiology of polyneuropathy in diabetes and prediabetes. Handb Clin Neurol (2014) 126:3–22. doi: 10.1016/B978-0-444-53480-4.00001-1 25410210

[4] American Diabetes Association. 11. Microvascular complications and foot care: standards of medical care in diabetes–2019. Diabetes Care (2019) 42:S124–38. doi: 10.2337/dc19-S011 30559237

[5] Scholz J Finnerup NB Attal N Aziz Q Baron R Bennett MI. The IASP classification of chronic pain for ICD-11: chronic neuropathic pain. Pain (2019) 160:53–9. doi: 10.1097/j.pain.0000000000001365 PMC6310153 30586071

[6] Tesfaye S Vileikyte L Rayman G Sindrup S Perkins B Baconja M. Consensus recommendations on diagnosis, assessment and management. Diabetes Metab Res Rev (2011) 27(7):629–38. doi: 10.1002/dmrr.1225 21695762

[7] Albers JW Pop-Busui R. Diabetic neuropathy: mechanisms, emerging treatments, and subtypes. Curr Neurol Neurosci Rep (2014) 14:473. doi: 10.1007/s11910-014-0473-5 24954624 PMC5084622

[8] Callaghan BC Xia R Reynolds E Banerjee M Burant C Rothberg A. Better diagnostic accuracy of neuropathy in obesity: a new challenge for neurologists. Clin Neurophysiol (2018) 129:654–62. doi: 10.1016/j.clinph.2018.01.003 PMC5808853 29414409

[9] Sharma S Vas P Rayman G. Small fiber neuropathy in diabetes polyneuropathy: is it time to change? J Diabetes Sci Technol (2022) 16:321–31. doi: 10.1177/1932296821996434 PMC8861803 33840265

[10] Armstrong DG Boulton AJM Bus SA. Diabetic foot ulcers and their recurrence. New Engl J Med (2017) 376:2367–75. doi: 10.1056/NEJMra1615439 28614678

[11] Burgess J Frank B Marshall A Khalil RS Ponirakis G Petropoulos IN. Early detection of diabetic peripheral neuropathy: a focus on small nerve fibres. Diagn (Basel) (2021) 11(2):165. doi: 10.3390/diagnostics11020165 PMC7911433 33498918

[12] Apfel SC Asbury AK Bril V Burns TM Campbell JN Chalk CH. Positive neuropathic sensory symptoms as endpoints in diabetic neuropathy trials. J Neurol Sci (2001) 189:3–5. doi: 10.1016/S0022-510X(01)00584-6 11596565

[13] Tesfaye S Stevens LK Stephenson JM Fuller JH Plater M Ionescu-Tirgoviste C. Prevalence of diabetic peripheral neuropathy and its relation to glycaemic control and potential risk factors: the EURODIAB IDDM complications study. Diabetologia (1996) 39:1377–84. doi: 10.1007/s001250050586 8933008

[14] Vas PRJ Green AQ Rayman G. Small fibre dysfunction, microvascular complications and glycaemic control in type 1 diabetes: a case-control study. Diabetologia (2012) 55:795–800. doi: 10.1007/s00125-011-2417-9 22193513

[15] Mizokami-Stout KR Li Z Foster NC Shah V Aleppo G McGill JB. The contemporary prevalence of diabetic neuropathy in type 1 diabetes: findings from the T1D exchange. Diabetes Care (2020) 43:806–12. doi: 10.2337/dc19-1583 PMC7085805 32029635

[16] Young M Boulton A MacLeod A Williams D Sonksen P. A multicentre study of the prevalence of diabetic peripheral neuropathy in the united kingdom hospital clinic population. Diabetologia (1993) 36:150–4. doi: 10.1007/BF00400697 8458529

[17] Rayman G Vas PR Baker N Taylor CG Gooday C Alder AI. The Ipswich touch test: a simple and novel method to identify inpatients with diabetes at risk of foot ulceration. Diabetes Care (2011) 34:1517–8. doi: 10.2337/dc11-0156 PMC3120164 21593300

[18] Sharma S Kerry C Atkins H Rayman G. The Ipswich touch test: a simple and novel method to screen patients with diabetes at home for increased risk of foot ulceration. Diabetes Med (2014) 31:1100–3. doi: 10.1111/dme.12450 24673517

[19] Martin CL Albers J Herman WH Cleary P Waberski B Greene DA. Neuropathy among the diabetes control and complications trial cohort 8 years after trial completion. Diabetes Care (2006) 29:340–4. doi: 10.2337/diacare.29.02.06.dc05-1549 PMC2622720 16443884

[20] Bril V Perkins BA. Validation of the Toronto clinical scoring system for diabetic polyneuropathy. Diabetes Care (2002) 25:2048–52. doi: 10.2337/diacare.25.11.2048 12401755

[21] Bril V Tomioka S Buchanan RA Perkins BA. Reliab validity modified Toronto Clin Neuropathy Score Diabetic sensorimotor polyneuropathy. Diabetes Med (2009) 26(3):240–6. doi: 10.1111/j.1464-5491.2009.02667.x PMC2871179 19317818

[22] Dyck PJ. Detection, characterization, and staging of polyneuropathy: assessed in diabetics. Muscle Nerve (1988) 11:21–32. doi: 10.1002/mus.880110106 3277049

[23] Singleton JR Bixby B Russell JW Feldman EL Peltier A Goldstein J. The Utah early neuropathy scale: a sensitive clinical scale for early sensory predominant neuropathy. J Peripher Nerv Syst (2008) 13:218–27. doi: 10.1111/j.1529-8027.2008.00180.x 18844788

[24] Haque F Reaz MBI Chowdhury MEH Shapiai MIB Malik RA Alhatou M. A machine learning-based severity prediction tool for the Michigan neuropathy screening instrument. Diagn (Basel) 13 (2023) 13(2):264. doi: 10.3390/diagnostics13020264 PMC9857736 36673074

[25] Bastyr EJ Price 3KL Bril V mTCNS. Group. Development and validity testing of the neuropathy total symptom score-6: questionnaire for the study of sensory symptoms of diabetic peripheral neuropathy. Clin Ther (2005) 27:1278–94. doi: 10.1016/j.clinthera.2005.08.002 16199253

[26] Spallone V Morganti R D’Amato C Greco C Cacciotti L Marfia GA. Validation of DN4 as a screening tool for neuropathic pain in painful diabetic polyneuropathy. Diabetes Med (2012) 29:578–85. doi: 10.1111/j.1464-5491.2011.03500.x 22023377

[27] Freynhagen R Baron R Gockel U Tolle TR. painDETECT: a new screening questionnaire to identify neuropathic components in patients with back pain. Curr Med Res Opin (2006) 22:1911–20. doi: 10.1185/030079906X132488 17022849

[28] Bouhassira D Attal N Fermanian J Alchaar H Gautron M Masquelier E. Development and validation of the neuropathic pain symptom inventory. Pain (2004) 108:248–57. doi: 10.1016/j.pain.2003.12.024 15030944

[29] Bonhof GJ Herder C Strom A Papanas N Roden M Ziegler D. Emerging biomarkers, tools, and treatments for diabetic polyneuropathy. Endocr Rev (2019) 40:153–92. doi: 10.1210/er.2018-00107 30256929

[30] Lee JA Halpern EM Lovblom LE Yeung E Bril V Perkins BA. Reliability and validity of a point-of-Care sural nerve conduction device for identification of diabetic neuropathy. PloS One (2014) 9:e86515. doi: 10.1371/journal.pone.0086515 24466129 PMC3899274

[31] Sharma S Vas PR Rayman G. Assessment of diabetic neuropathy using a point-of-care nerve conduction device shows significant associations with the LDIFLARE method and clinical neuropathy scoring. J Diabetes Sci Technol (2015) 9:123–31. doi: 10.1177/1932296814551044 PMC4495534 25231114

[32] Selvarajah D Cash T Davies J Sankar A Rao G Grieg M. SUDOSCAN: a simple, rapid, and objective method with potential for screening for diabetic peripheral neuropathy. PloS One (2015) 10:e0138224. doi: 10.1371/journal.pone.0138224 26457582 PMC4601729

[33] Cabre JJ Mur T Costa B Barrio F Lopez-Moya C Sagarra R. Feasibility and effectiveness of electrochemical dermal conductance measurement for the screening of diabetic neuropathy in primary care. decoding study (Dermal electrochemical conductance in diabetic neuropathy). J Clin Med (2019) 8. doi: 10.3390/jcm8050598 PMC6572367 31052426

[34] Bril V Kojic J Ngo M Clark K. Comparison of a neurothesiometer and vibration in measuring vibration perception thresholds and relationship to nerve conduction studies. Diabetes Care (1997) 20:1360–2. doi: 10.2337/diacare.20.9.1360 9283779

[35] van Deursen RW Sanchez MM Derr JA Becker MB Ulbrecht JS Cavanagh PR. Vibration perception threshold testing in patients with diabetic neuropathy: ceiling effects and reliability. Diabetes Med (2001) 18:469–75. doi: 10.1046/j.1464-5491.2001.00503.x 11472466

[36] Abbott CA Malik RA van Ross ER Kulkarni J Boulton AJ. Prevalence and characteristics of painful diabetic neuropathy in a large community-based diabetic population in the U.K. Diabetes Care (2011) 34:2220–4. doi: 10.2337/dc11-1108 PMC3177727 21852677

[37] Perkins B Bril A. Electrophysiologic testing in diabetes neuropathy. In: Zochodne DM, editor. Diabetes & the nervous system. New York: Elsevier (2014). p. 235–48.

[38] Dyck PJ Litchy WJ Lehman KA Hokanson JL Low PA O’Brien PC. Variables influencing neuropathic endpoints: the Rochester diabetic neuropathy study of healthy subjects. Neurology (1995) 45:1115–21. doi: 10.1212/WNL.45.6.1115 7783874

[39] Said G Baudoin D Toyooka K. Sensory loss, pains, motor deficit and axonal regeneration in length-dependent diabetic polyneuropathy. J Neurol (2008) 255:1693–702. doi: 10.1007/s00415-008-0999-z 18825430

[40] Malik RA Tesfaye S Newrick PG Walker D Rajbhandari SM Siddique I. Sural nerve pathology in diabetic patients with minimal but progressive neuropathy. Diabetologia (2005) 48:578–85. doi: 10.1007/s00125-004-1663-5 15729579

[41] Chao CC Hsieh SC Yang WS Lin YH Lin WM Tai TY. Glycemic control is related to the severity of impaired thermal sensations in type 2 diabetes. Diabetes Metab Res Rev (2007) 23:612–20. doi: 10.1002/dmrr.734 17354257

[42] Vas PR Rayman G. Validation of the modified LDIFlare technique: a simple and quick method to assess c-fiber function. Muscle Nerve (2013) 47:351–6. doi: 10.1002/mus.23532 23169592

[43] Sharma S Tobin V Vas PRJ Malik RA Rayman G. The influence of age, anthropometric and metabolic variables on LDIFLARE and corneal confocal microscopy in healthy individuals. PloS One (2018) 13:e0193452. doi: 10.1371/journal.pone.0193452 29518115 PMC5843248

[44] Sharma S Tobin V Rayman G. A prospective study of small fibre structure and function in newly diagnosed type 1 diabetes. presented at the 76th scientific sessions of the American diabetes association (New Orleans, USA. June 10-14, 2016). Diabetes (2016) 65:A101–69.

[45] Inceu GV Veresiu IA. Measurement of current perception thresholds using the Neurometer((R)) - applicability in diabetic neuropathy. Clujul Med (2015) 88:449–52. doi: 10.15386/cjmed-491 PMC4689234 26733741

[46] Ruscheweyh R Emptmeyer K Putzer D Kropp P Marziniak M. Reproducibility of contact heat evoked potentials (CHEPs) over a 6 months interval. Clin Neurophysiol (2013) 124:2242–7. doi: 10.1016/j.clinph.2013.05.003 23746497

[47] Vallbo AB. Microneurography: how it started and how it works. J Neurophysiol (2018) 120:1415–27. doi: 10.1152/jn.00933.2017 29924706

[48] La Cesa S Di Stefano G Leone C Pepe A Galosi E Alu F. Skin denervation does not alter cortical potentials to surface concentric electrode stimulation: a comparison with laser evoked potentials and contact heat evoked potentials. Eur J Pain (2018) 22:161–9. doi: 10.1002/ejp.1112 28898491

[49] Shimada H Kihara M Kosaka S Ikeda H Kawabata K Tsutada T. Comparison of SSR and QSART in early diabetic neuropathy–the value of length-dependent pattern in QSART. Auton Neurosci (2001) 92:72–5. doi: 10.1016/S1566-0702(01)00287-9 11570706

[50] Sun PC Lin HD Jao SH Chan RC Kao MJ Cheng CK. Thermoregulatory sudomotor dysfunction and diabetic neuropathy develop in parallel in at-risk feet. Diabetes Med (2008) 25:413–8. doi: 10.1111/j.1464-5491.2008.02395.x 18341593

[51] Lin X Chen C Liu Y Peng Y Chen Z Huang H. Peripheral nerve conduction and sympathetic skin response are reliable methods to detect diabetic cardiac autonomic neuropathy. Front Endocrinol (Lausanne) (2021) 12:709114. doi: 10.3389/fendo.2021.709114 34621241 PMC8490774

[52] Papanas N Paschos P Papazoglou D Papatheodorou K Paletas K Maltezos E. Accuracy of the neuropad test for the diagnosis of distal symmetric polyneuropathy in type 2 diabetes. Diabetes Care (2011) 34:1378–82. doi: 10.2337/dc10-2205 PMC3114325 21505209

[53] Lauria G Bakkers M Schmitz C Lombardi R Penza P Devigili G. Intraepidermal nerve fiber density at the distal leg: a worldwide normative reference study. J Peripher Nerv Syst (2010) 15:202–7. doi: 10.1111/j.1529-8027.2010.00271.x 21040142

[54] Chambers AR Song K. Sural nerve biopsy. Treasure Island (FL: StatPearls (2022). 31869109

[55] Petropoulos IN Ponirakis G Khan A Gad H Almuhannadi H Brines M. Corneal confocal microscopy: ready for prime time. Clin Exp Optometry (2019) 103(3):265–77. doi: 10.1111/cxo.12887 30834591

[56] Pritchard N Edwards K Russell AW Perkins BA Malik RA Efron N. Corneal confocal microscopy predicts 4-year incident peripheral neuropathy in type 1 diabetes. Diabetes Care (2015) 38:671–5. doi: 10.2337/dc14-2114 25573881

[57] Dineen J Freeman R. Autonomic neuropathy. Semin Neurol (2015) 35:458–68. doi: 10.1055/s-0035-1558983 26502768

[58] Spallone V. Update on the impact, diagnosis and management of cardiovascular autonomic neuropathy in diabetes: what is defined, what is new, and what is unmet. Diabetes Metab J (2019) 43:3–30. doi: 10.4093/dmj.2018.0259 30793549 PMC6387879

[59] Greco C Di Gennaro F D’Amato C Morganti R Corradini D Sun A. Validation of the composite autonomic symptom score 31 (COMPASS 31) for the assessment of symptoms of autonomic neuropathy in people with diabetes. Diabetes Med (2017) 34:834–8. doi: 10.1111/dme.13310 27990686

[60] Sloan G Alam U Selvarajah D Tesfaye S. The treatment of painful diabetic neuropathy. Curr Diabetes Rev (2022) 18:e070721194556. doi: 10.2174/1573399817666210707112413 34238163

[61] Gandhi R Selvarajah D Sloan G Greig M Wilkinson ID Shaw PJ. Preservation of thalamic neuronal function may be a prerequisite for pain perception in diabetic neuropathy: a magnetic resonance spectroscopy study. Front Pain Res (Lausanne) (2022) 3:1086887. doi: 10.3389/fpain.2022.1086887 36688084 PMC9852821

[62] C. American Diabetes Association Professional Practice. 4. comprehensive medical evaluation and assessment of comorbidities: standards of medical care in diabetes-2022. Diabetes Care (2022) 45:S46–59. doi: 10.2337/dc22-S004 PMC8935396 34964869

[63] Sharma S Cross J Rayman G. 83-OR: the LDIFLAREMethod predicts the development of incipient diabetes polyneuropathy: results of the five-year longitudinal Ipswich NeuroDiab study. Diabetes (2022) 71. doi: 10.2337/db22-83-OR

[64] Tavakoli M Ferdousi M Petropoulos IN Morris J Pritchard N Zhivov A. Normative values for corneal nerve morphology assessed using corneal confocal microscopy: a multinational normative data set. Diabetes Care (2015) 38:838–43. doi: 10.2337/dc14-2311 PMC4407754 25633665

[65] McArthur JC Stocks EA Hauer P Cornblath DR Griffin JW. Epidermal nerve fiber density: normative reference range and diagnostic efficiency. Arch Neurol (1998) 55:1513–20. doi: 10.1001/archneur.55.12.1513 9865794

[66] Pop-Busui R Roberts L Pennathur S Kretzler M Brosius FC Feldman EL. The management of diabetic neuropathy in CKD. Am J Kidney Dis (2010) 55:365–85. doi: 10.1053/j.ajkd.2009.10.050 PMC4007054 20042258

[67] Look ARG. Effects of a long-term lifestyle modification programme on peripheral neuropathy in overweight or obese adults with type 2 diabetes: the look AHEAD study. Diabetologia (2017) 60:980–8. doi: 10.1007/s00125-017-4253-z PMC5423967 28349174

[68] Callaghan BC Reynolds EL Banerjee M Akinci G Chant E Villegas-Umana E. Dietary weight loss in people with severe obesity stabilizes neuropathy and improves symptomatology. Obes (Silver Spring) (2021) 29:2108–18. doi: 10.1002/oby.23246 PMC8612943 34747574

[69] Martin CL Albers JW Pop-Busui R D.E.R. Group. Neuropathy and related findings in the diabetes control and complications trial/epidemiology of diabetes interventions and complications study. Diabetes Care (2014) 37:31–8. doi: 10.2337/dc13-2114 PMC3868000 24356595

[70] Gaede P Lund-Andersen H Parving HH Pedersen O. Effect of a multifactorial intervention on mortality in type 2 diabetes. New Engl J Med (2008) 358:580–91. doi: 10.1056/NEJMoa0706245 18256393

[71] Boussageon R Bejan-Angoulvant T Saadatian-Elahi M Lafont S Bergeonneau C Kassai B. Effect of intensive glucose lowering treatment on all cause mortality, cardiovascular death, and microvascular events in type 2 diabetes: meta-analysis of randomised controlled trials. BMJ 343 (2011) 343:d4169. doi: 10.1136/bmj.d4169 PMC3144314 21791495

[72] Ziegler D Tesfaye S Spallone V Gurieva I Al Kaabi J Mankovsky B. Screening, diagnosis and management of diabetic sensorimotor polyneuropathy in clinical practice: international expert consensus recommendations. Diabetes Res Clin Pract (2022) 186:109063. doi: 10.1016/j.diabres.2021.109063 34547367

[73] Tesfaye S Selvarajah D. The eurodiab study: what has this taught us about diabetic peripheral neuropathy? Curr Diabetes Rep (2009) 9:432–4. doi: 10.1007/s11892-009-0070-1 19954687

[74] Balakumar P Rohilla A Krishan P Solairaj P Thangathirupathi A. The multifaceted therapeutic potential of benfotiamine. Pharmacol Res (2010) 61:482–8. doi: 10.1016/j.phrs.2010.02.008 20188835

[75] Papanas N Ziegler D. Efficacy of alpha-lipoic acid in diabetic neuropathy. Expert Opin Pharmacother (2014) 15:2721–31. doi: 10.1517/14656566.2014.972935 25381809

[76] Ziegler D Low PA Litchy WJ Boulton AJ Vinik AI Freeman R. Efficacy and safety of antioxidant treatment with alpha-lipoic acid over 4 years in diabetic polyneuropathy: the NATHAN 1 trial. Diabetes Care (2011) 34:2054–60. doi: 10.2337/dc11-0503 PMC3161301 21775755

[77] Ziegler D Movsesyan L Mankovsky B Gurieva I Abylaiuly Z Strokov I. Treatment of symptomatic polyneuropathy with actovegin in type 2 diabetic patients. Diabetes Care (2009) 32:1479–84. doi: 10.2337/dc09-0545 PMC2713653 19470838

[78] Hotta N Akanuma Y Kawamori R Matsuoka K Oka Y Shichiri M. Long-term clinical effects of epalrestat, an aldose reductase inhibitor, on diabetic peripheral neuropathy: the 3-year, multicenter, comparative aldose reductase inhibitor-diabetes complications trial. Diabetes Care (2006) 29:1538–44. doi: 10.2337/dc05-2370 16801576

[79] El-Nahas MR Elkannishy G Abdelhafez H Elkhamisy ET El-Sehrawy AA. Oral alpha lipoic acid treatment for symptomatic diabetic peripheral neuropathy: a randomized double-blinded placebo-controlled study. Endocr Metab Immune Disord Drug Targets (2020) 20:1531–4. doi: 10.2174/1871530320666200506081407 32370731

[80] Tesfaye S Sloan G Petrie J White D Bradburn M Young T. Optimal pharmacotherapy pathway in adults with diabetic peripheral neuropathic pain: the OPTION-DM RCT. Health Technol Assess (2022) 26:1–100. doi: 10.3310/RXUO6757 PMC9589396 36259684

[81] Tesfaye S Wilhelm S Lledo A Schacht A Tolle T Bouhassira D. Duloxetine and pregabalin: high-dose monotherapy or their combination? the “COMBO-DN study”–a multinational, randomized, double-blind, parallel-group study in patients with diabetic peripheral neuropathic pain. Pain (2013) 154:2616–25. doi: 10.1016/j.pain.2013.05.043 23732189

[82] Dy SM Bennett WL Sharma R Zhang A Waldfogel JM Nesbit SA. Preventing complications and treating symptoms of diabetic peripheral neuropathy [Internet]. Rockville (MD): Agency for Healthcare Research and Quality (US). (2017). Report No.: 17-EHC005-EF. 28749633

[83] Snedecor SJ Sudharshan L Cappelleri JC Sadosky A Mehta S Botteman M. Systematic review and meta-analysis of pharmacological therapies for painful diabetic peripheral neuropathy. Pain Pract (2014) 14:167–84. doi: 10.1111/papr.12054 23534696

[84] Liampas A Rekatsina M Vadalouca A Paladini A Varrassi G Zis P. Pharmacological management of painful peripheral neuropathies: a systematic review. Pain Ther (2021) 10:55–68. doi: 10.1007/s40122-020-00210-3 33145709 PMC8119529

[85] Griebeler ML Morey-Vargas OL Brito JP Tsapas A Wang Z Carranza Leon BG. Pharmacologic interventions for painful diabetic neuropathy: an umbrella systematic review and comparative effectiveness network meta-analysis. Ann Internal Med (2014) 161:639–49. doi: 10.7326/M14-0511 25364885

[86] Simpson DM Robinson-Papp J Van J Stoker M Jacobs H Snijder RJ. Capsaicin 8% patch in painful diabetic peripheral neuropathy: a randomized, double-blind, placebo-controlled study. J Pain (2017) 18:42–53. doi: 10.1016/j.jpain.2016.09.008 27746370

[87] Pollard EM Lamer TJ Moeschler SM Gazelka HM Hooten WM Bendel MA. The effect of spinal cord stimulation on pain medication reduction in intractable spine and limb pain: a systematic review of randomized controlled trials and meta-analysis. J Pain Res (2019) 12:1311–24. doi: 10.2147/JPR.S186662 PMC6502439 31118751

[88] Petersen EA Stauss TG Scowcroft JA Brooks ES White JL Sills SM. Effect of high-frequency (10-kHz) spinal cord stimulation in patients with painful diabetic neuropathy: a randomized clinical trial. JAMA Neurol (2021) 78:687–98. doi: 10.1001/jamaneurol.2021.0538 PMC8022268 33818600

[89] Amato Nesbit S Sharma R Waldfogel JM Zhang A Bennett WL Yeh HC. Non-pharmacologic treatments for symptoms of diabetic peripheral neuropathy: a systematic review. Curr Med Res Opin (2019) 35:15–25. doi: 10.1080/03007995.2018.1497958 30114983

